# Sequencing and Analysis of the *Pseudomonas fluorescens* GcM5-1A Genome: A Pathogen Living in the Surface Coat of *Bursaphelenchus xylophilus*

**DOI:** 10.1371/journal.pone.0141515

**Published:** 2015-10-30

**Authors:** Kai Feng, Ronggui Li, Yingnan Chen, Boguang Zhao, Tongming Yin

**Affiliations:** 1 Co-Innovation Center for Sustainable Forestry in Southern China, College of Forestry, Nanjing Forestry University, Nanjing, 210037, China; 2 Department of Biology, Qingdao University, Qingdao, 266071, China; 3 College of Forestry, Nanjing Forestry University, Nanjing, 210037, China; Oklahoma State University, UNITED STATES

## Abstract

It is known that several bacteria are adherent to the surface coat of pine wood nematode (*Bursaphelenchus xylophilus)*, but their function and role in the pathogenesis of pine wilt disease remains debatable. The *Pseudomonas fluorescens* GcM5-1A is a bacterium isolated from the surface coat of pine wood nematodes. In previous studies, GcM5-1A was evident in connection with the pathogenicity of pine wilt disease. In this study, we report the *de novo* sequencing of the GcM5-1A genome. A 600-Mb collection of high-quality reads was obtained and assembled into sequence contigs spanning a 6.01-Mb length. Sequence annotation predicted 5,413 open reading frames, of which 2,988 were homologous to genes in the other four sequenced *P*. *fluorescens* isolates (SBW25, WH6, Pf0-1 and Pf-5) and 1,137 were unique to GcM5-1A. Phylogenetic studies and genome comparison revealed that GcM5-1A is more closely related to SBW25 and WH6 isolates than to Pf0-1 and Pf-5 isolates. Towards study of pathogenesis, we identified 79 candidate virulence factors in the genome of GcM5-1A, including the *Alg*, *Fl*, *Waa* gene families, and genes coding the major pathogenic protein *fliC*. In addition, genes for a complete T3SS system were identified in the genome of GcM5-1A. Such systems have proved to play a critical role in subverting and colonizing the host organisms of many gram-negative pathogenic bacteria. Although the functions of the candidate virulence factors need yet to be deciphered experimentally, the availability of this genome provides a basic platform to obtain informative clues to be addressed in future studies by the pine wilt disease research community.

## Introduction

*Pseudomonas fluorescens* are Gram-negative bacteria that have diverse lifestyles and versatile metabolisms. These bacteria are found in decaying and living plants, soil, and water. Some *P*. *fluorescens* isolates benefit plants by suppressing pathogens, aiding in nutrient absorption, and degrading environmental pollutants [1]. Other isolates produce compounds that negatively affect the plant’s growth [2].

To date, the genomes of four isolates of *P*. *fluorescens* have been sequenced and are publicly available, including SBW25, WH6, Pf0-1, and Pf-5 [3–5]. The published genomes revealed that *P*. *fluorescens* has an open pan-genome of approximately 6 to 7 Mb. In addition to the core genes, each isolate possesses 1000 to 1500 unique genes. Previous phylogenetic studies categorized the four sequenced *P*. *fluorescens* isolates into two distinct clusters: SBW25 and WH6 formed one cluster, and Pf0-1 and Pf-5 formed a second cluster. In these sequenced isolates, Pf-5 and SBW25 are rhizosphere bacteria that promote plant growth and suppress plant pathogens [3] [6]. WH6 produces a chemical compound called the germination-arrest factor (GAF) that specifically and irreversibly blocks the germination of the seeds of a large number of grassy weed species without significantly affecting the growth of established seedlings or mature plants [7]. And Pf0-1 is a bacterium that is well adapted to the soil environment and contributes significantly to the turnover of organic matter [5].

Another isolate of *P*. *fluorescens*, GcM5-1A, was reported to increase egg production, developmental rate, body length and diameter of both male and female pine wood nematodes (PWN) [8]. GcM5-1A is one of the main bacteria isolated from the surface coat of *Bursaphelenchus xylophilus* (the nematode causing pine wilt disease), which has been implicated in connection with the pathogenesis of pine wilt disease (PWD) in several previous studies [9–12]. The devastating pine wilt disease spreads among pine trees in Asian countries, especially in Japan, China and Korea. This disease is caused by PWN (whose genome has been sequenced in 2011 [13]), and is transferred by pine sawyer beetles (*Monochamus alternatus*) [14]. PWN can reproduce quickly in the sapwood of the susceptible pine species, which causes wilting and death of the host in a short period of time. Recently, this disease has also been observed in European countries [15], and is gradually becoming a threat to pine forests worldwide.

The pathogenesis of PWD remains debatable. PWN was previously recognized as the only pathogen that caused this disease [16]. However, it was reported that PWN lost pathogenicity after surface sterilization [17], which led to the hypothesis that the pathogenicity might be a combined effect of the PWN and the microbes adherent to their surface coat. Various evidence supported this hypothesis. For instance, Shinya *et al*.’s study indicated that the surface coat of PWN could protect the microbes and was essential for the infection of pine trees by PWN [9]. In addition, Guo *et al*. reported that *P*. *fluorescens* GcM5-1A, isolated from the surface coat of PWN, could produce a flagellin, namely *fliC*, that was able to increase populations of pine wood nematodes and their associated bacteria [10]. It was also found that *fliC* could induce the death of *Pinus thunbergii* suspension cells in 24 h [11], and Xu *et al*. proved that *fliC* increased damage to the host pine through enhancing the oxidative stress [12].

Although several studies have been carried out testing the connection of the PWN surface coat microbes with the pathogenicity of PWD, their relationship remain debatable [18], and the underlying mechanisms need to be deciphered at the molecular level. Many virulence factors may play a role in the infection process. For instance, it was found that many gram-negative pathogenic bacteria employed a type III secretion system (T3SS) to subvert and colonize their host organisms. The T3SS injects effector proteins directly into the cytosol of eukaryotic cells, and thus allows the manipulation of host cellular activities to the benefit of the pathogen [19]. T3SS provides a unique virulence mechanism to infect host cells [20]. Among the published genomes of the *P*. *fluorescens* isolates, a complete T3SS was only identified in WH6 [4], which produces the GAF herbicide that helps to control grassy weeds [21]. Whether GcM5-1A possesses a complete T3SS remains unknown. Besides the T3SS system, other candidate virulence factors may exist and need to be addressed in understanding the role of PWN surface coat microbes in connection with the pathogenicity of PWD at the molecular level.

Taken together, the perspectives of this study are to: 1) sequence the genome of GcM5-1A to provide a basic platform for the pine wilt disease research community; 2) compare the genome of GcM5-1A and study the phylogenetic relationship of GcM5-1A with that of the other four sequenced *P*. *fluorescens* isolates at genome-wide level; and 3) detect the candidate virulence factors, including the T3SS system, based on sequence annotation, to provide clues to be addressed in future studies.

## Results and Discussion

### Genome sequencing and assembling

**Fig 1:**
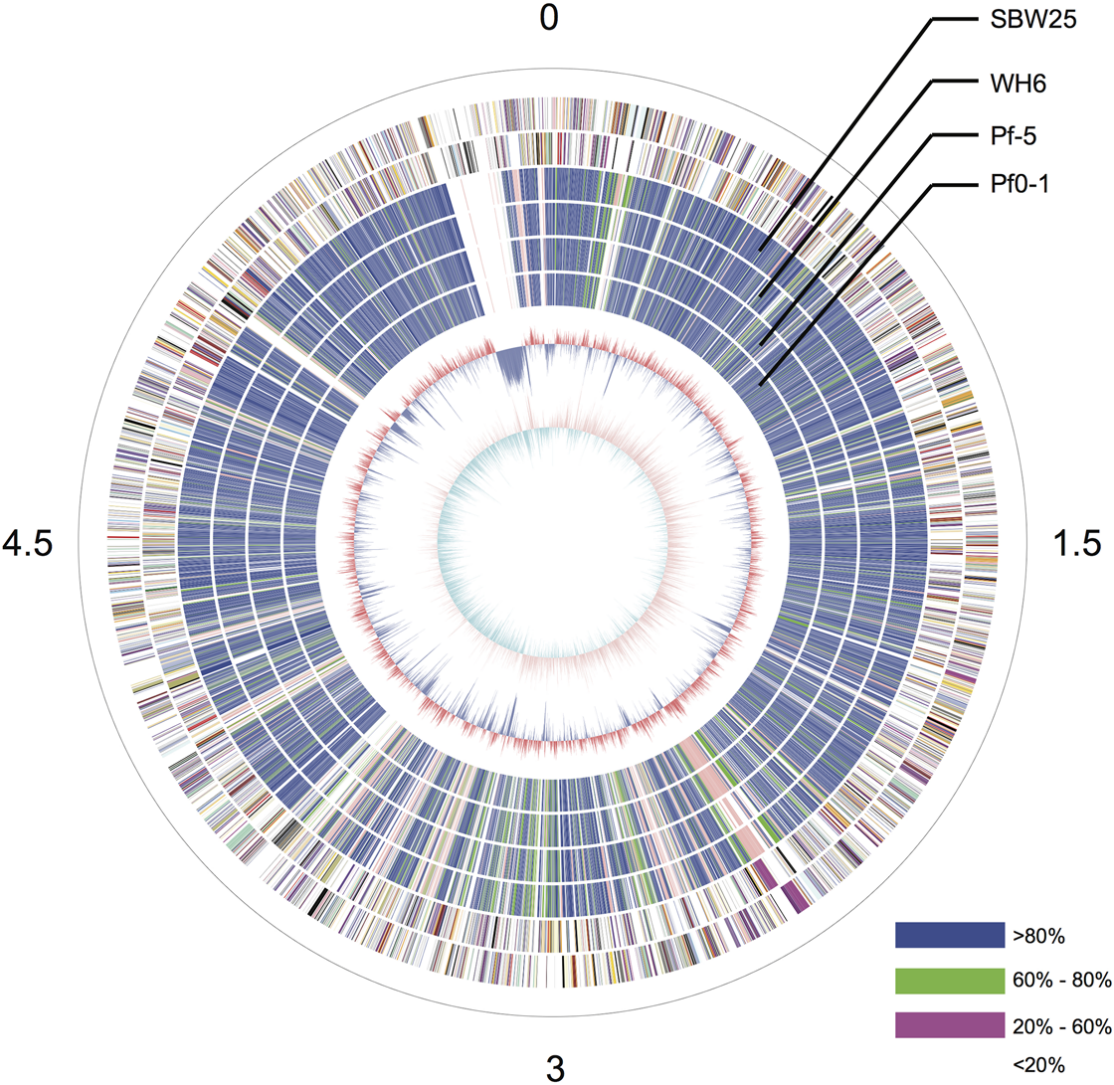
Circular graph of *P*. *fluorescens* GcM5-1A. From the outer to the innermost circle, circle 1 presents the physical coordinates, in 100,000 bp per interval, as defined by the black sticks. The red sticks on circle 1 indicate the sites of the physical gaps. Circles 2 and 3 depict the CDS on the positive and negative strands. Circles 4, 5, 6 and 7 depict the orthologous similarity between GcM5-1A and WH6, between GcM5-1A and SBW25, between GcM5-1A and Pf-5, and between GcM5-1A and Pf0-1, respectively. Different colors indicate different similarity levels. Blue indicates similarity greater than 80%. Green indicates similarity within the range of 60 to 80%. Pink indicates similarity within the range of 20 to 60%. White reflects similarity less than 20%. Circle 8 indicates the GC content (red > 60.5%, blue < 60.5%). Circle 9 depicts the GC-skew (purple > 0, cyan < 0).

The GcM5-1A genome was sequenced using a 454 GS-FLX sequencer with a GS titanium XLR70 kit (Roche Inc.), which resulted in 1,534,199 sequencing reads with an average length of 409 bp. The total throughput exceeded 600 Mb. The genome sizes of the four sequenced *P*. *fluorescens* isolates ranged from 6.27 to 7.07 Mb. Using this range as a baseline, the sequencing depth of GcM5-1A was estimated to be approximately 100X. After filtering with the Newbler *de novo* Assembler (Roche Inc.), 98.23% of the total reads were selected and used in the final assembly. As a result, the genome assembly generated 263 contigs greater than 100 bp, among which 79 contigs were greater than 500 bp. The final assembly covered 6.01 Mb. Subsequently, all of the contigs were submitted to BLAST against the nr database using BLASTX [22]. No contigs exhibited high similarity with genes from organisms outside the bacterial kingdom, except for contig24, which shared a highly homologous DNA fragment with the *Pseudomonas* phage phiPsa374, with 73% sequence identity. The homologous fragment of contig24 covered 34% of the genome of phiPsa374. Therefore, we placed contig24 at the terminus of chromosome replication of the established circular graph (**Fig 1**). The sequence alignment revealed that the contig shared almost no sequence similarity with any of the other *P*. *fluorescens* isolates. Further analysis revealed that contig24 had a size and GC content similar to those of several *Pseudomonas* phages. We hypothesized that contig24 might represent a horizontally transferred phage sequence that diverged significantly from the archetype during the evolutionary process or an exogenous DNA segment from an unknown phage.

To order the obtained contigs, a high-quality reference genome was required. As revealed by BLASTX, GcM5-1A exhibited higher similarity with SBW25 and WH6 compared with Pf0-1 and Pf-5. Compared with SBW25, WH6 was incompletely sequenced [4]. Therefore, the genome assembly of SBW25 was selected as a reference to order the obtained sequence contigs. All contigs greater than 1 kb were ordered in line with the reference genome by the Mauve Aligner [23]. There were 188 contigs smaller than 1 kb. Together with contig24, these contigs only accounted for 2.1% of the GcM5-1A genome. Among the ordered contigs, contig4 contained the gene which is responsible for initiation of replication; thus, it was marked as the starting point of the GcM5-1A genome.

### Genome comparison of the sequenced *P*. *fluorescens* isolates

**Fig 2:**
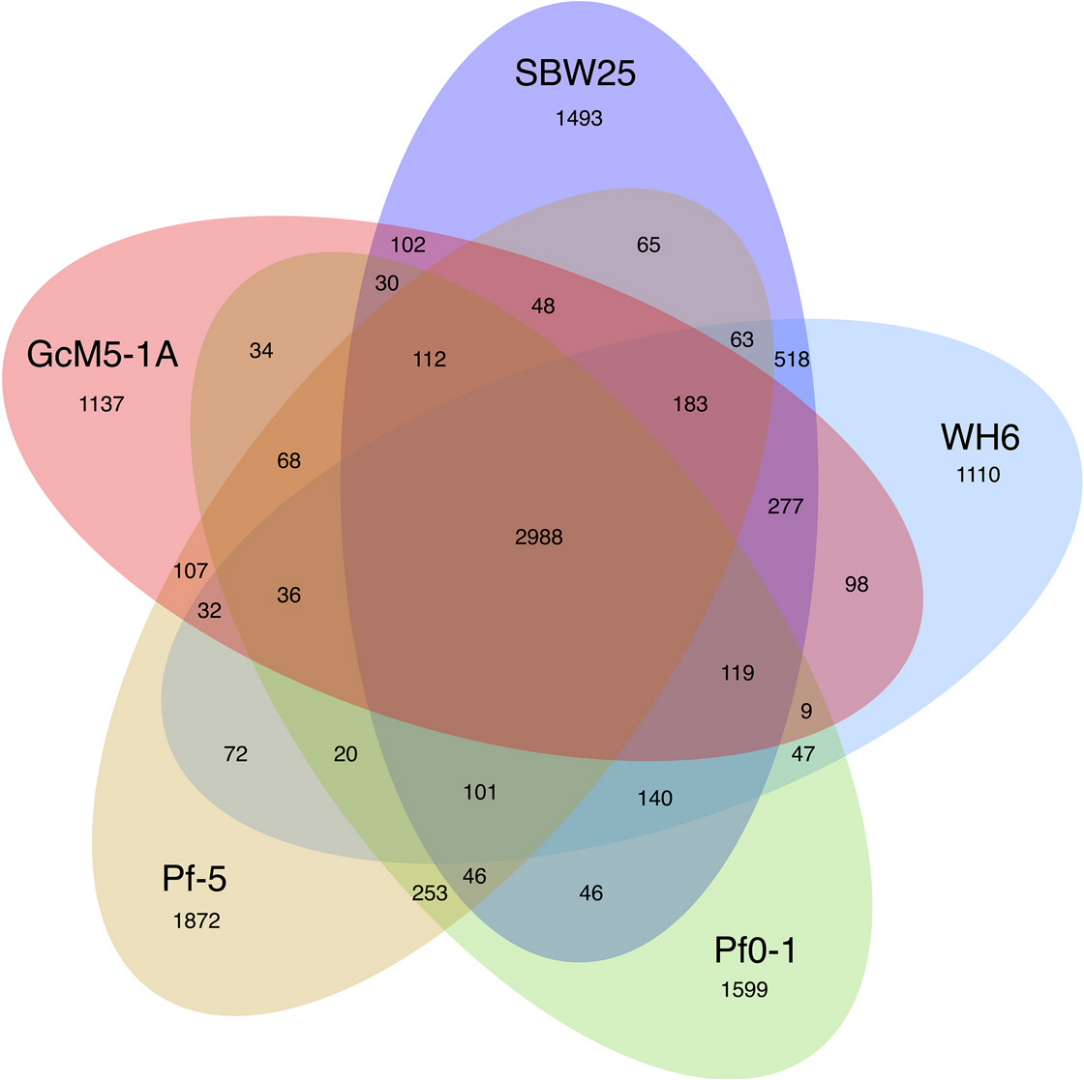
Shared and unique genes in each of the sequenced *P*. *fluorescens* isolates.

**Fig 3:**
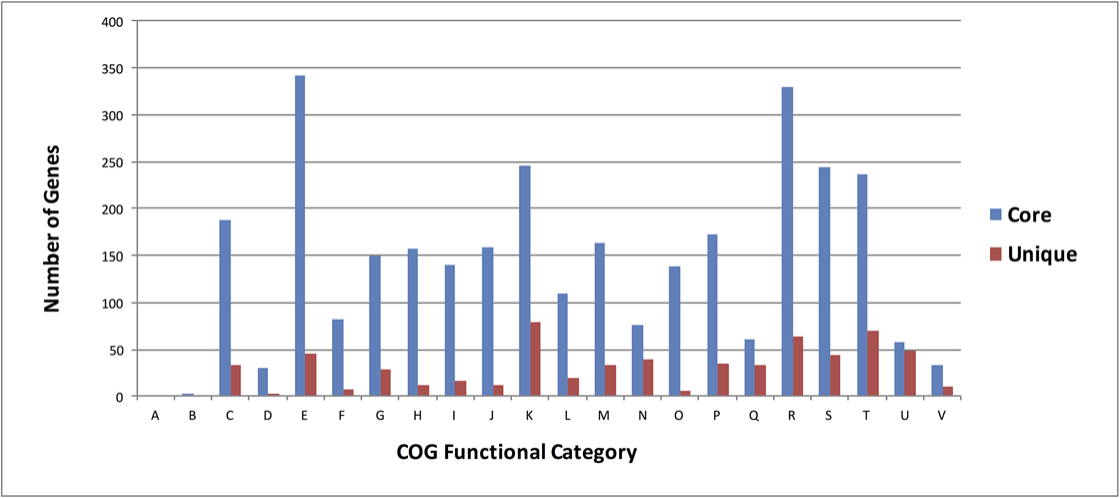
Functional classification of core and unique genes of GcM5-1A. A: RNA processing and modification; B: Chromatin structure and dynamics; C: Energy production and conversion; D: Cell cycle control, cell division, chromosome partitioning; E: Amino acid transport and metabolism; F: Nucleotide transport and metabolism; G: Carbohydrate transport and metabolism; H: Coenzyme transport and metabolism; I: Lipid transport and metabolism; J: Translation, ribosomal structure and biogenesis; K: Transcription; L: Replication, recombination and repair; M: Cell wall/membrane/envelope biogenesis; N: Cell motility; O: Posttranslational modification, protein turnover, chaperones; P: Inorganic ion transport and metabolism; Q: Secondary metabolites biosynthesis, transport and catabolism; R: General function prediction only; S: Function unknown; T: Signal transduction mechanisms; U: Intracellular trafficking, secretion, and vesicular transport; V: Defense mechanisms

A total of 5,413 coding sequences (CDSs) were predicted in the genome of GcM5-1A. The sequences were annotated based on similarity with the nr database. According to the features of the GcM5-1A genome (**Table 1**), there were fewer predicted CDSs noted in GcM5-1A than in any of the other four sequenced *P*. *fluorescens* isolates. In contrast, the GcM5-1A genome contained an increased number of tRNA compared with any other *P*. *fluorescens* isolates. We used OrthoMCL [24] to search for the orthologous groups, and 2,988 orthologous groups were found **(Fig 2**). These orthologous groups formed the core gene set of *P*. *fluorescens*; the Clusters of Orthologous Groups (COG) database was then used for functional annotation of the core gene set and unique genes of GcM5-1A [25]. The annotation results (**Fig 3**) were similar to those of WH6 [4]. The core gene set accounted for 50.8%, 50.4%, 52.1%, and 48.7% of the total genes in the SBW25, WH6, Pf0-1, and Pf-5 genomes, respectively. The percentage of shared orthologous groups between GcM5-1A and SBW25 was 70%, between GcM5-1A and WH6 was 68%, between GcM5-1A and Pf0-1 was 62%, and between GcM5-1A and Pf-5 was 65%. By contrast, it was 82% between the *Pseudomonas syringae* pv. *syringae* B728a and *P*. *syringae* pv. *tomato* DC3000 [26], and it was at least 86% among the 5 isolates (PA2192, C3719, PA01, PA14 and PACS5) of *P*. *aeruginosa* [27]. Compared with the increased percentage of shared orthologous groups between the isolates of *P*. *syringae* and *P*. *aeruginosa*, the lower percentage of shared orthologous groups between isolates of *P*. *fluorescens* indicated that the genomes of *P*. *fluorescens* isolates had undergone a heavier gene reshuffling than other *Pseudomonas* species.

**Table 1:** Genome features of GcM5-1A and other sequenced *P*. *fluorescens* isolates.

	GcM5-1A	WH6	SBW25	Pf-5	Pf0-1
**Genome Size**	6.01 Mb	6.27 Mb	6.72 Mb	7.07 Mb	6.44 Mb
**GC content (%)**	60.5	60.6	60.5	63.3	60.5
**Predicted CDSs**	5,413	5,876	5,921	6,138	5,736
**Avg. CDS length (bp)**	949	951	1,000	1,020	1,006
**Coding percentage**	85.5	89.2	88.1	88.5	89.6
**rRNA**	4	4	5	5	6
**tRNA (pseudo)**	78 (2)	53	66	71	73

### Phylogenetic and syntenic analyses

**Fig 4:**
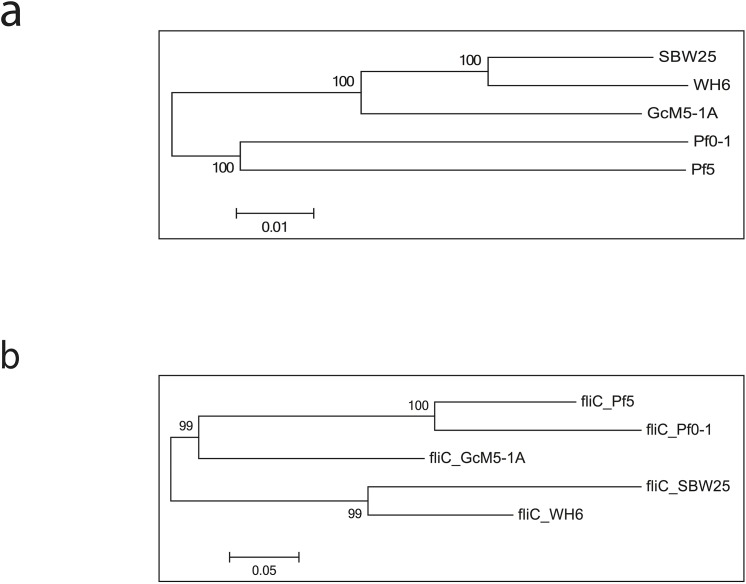
Phylogenetic tree of the five sequenced *P*. *fluorescens* isolates and the phylogenetic tree of *fliC* genes. (a) the phylogenetic tree was generated based on the multiple alignments of amino acid sequences of 2,935 single-copy orthologous groups. (b) Gene tree constructed for the 5 sequenced *P*. *fluorescens* isolates based on the amino acid sequence of the *fliC* gene.

To investigate the phylogenetic relationship among the five sequenced *P*. *fluorescens* genomes, 2,935 single-copy orthologous groups were used to generate a multiple alignment using MUSCLE [28] with default parameters. A phylogenetic tree was then constructed using MEGA5 [29] with the neighborhood-joining method and the bootstrap parameter set to 500. Previous studies categorized the *P*. *fluorescens* isolates into two distinct clades. SBW25 and WH6 formed one clade, and Pf0-1 and Pf-5 formed the second clade. In this study, GcM5-1A was evident in the SBW25 and Wh6 clade (**Fig 4A**).

**Fig 5:**
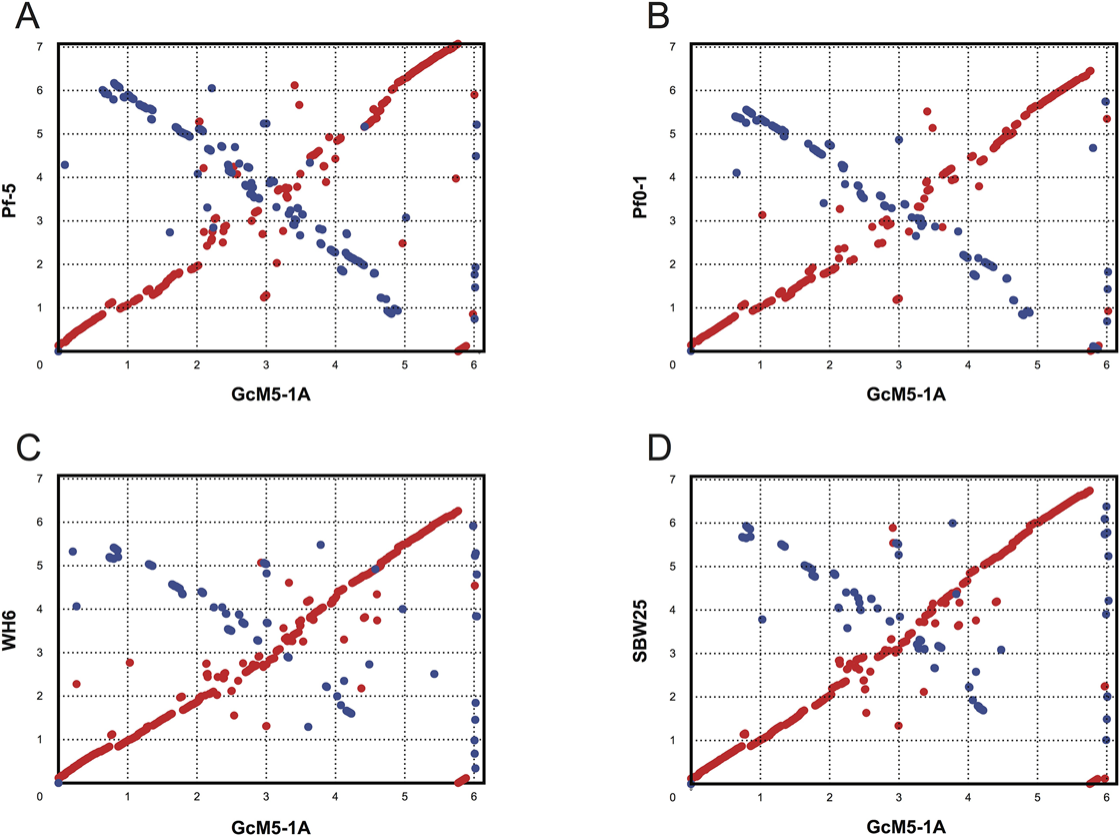
Synteny plots of GcM5-1A and the other sequenced *P*. *fluorescens* isolates. Genomes of the *P*. *fluorescens* isolates Pf-5 (A), Pf0-1 (B), WH6 (C) and SBW25 (D) were compared with the WH6 genome sequence. Genome scales are indicated in 1-Mb increments.

Long-range synteny of GcM5-1A with other *P*. *fluorescens* isolates appeared more often at the origin and terminus of chromosome replication of the genome. Compared with Pf0-1 and Pf-5, fewer outliers were noted between the genome of GcM5-1A and those of SBW25 and WH6, indicating that the genome of GcM5-1A shares higher colinearity with that of the latter than the former (**Fig 5**). Thus, GcM5-1A was more closely related to SBW25 and WH6 than to Pf0-1 and Pf-5, which was consistent with the results obtained from the phylogenetic tree. Given that the genome assembly of GcM5-1A was ordered using SBW25 as reference, the synteny between GcM5-1A and SBW25 was expected to be better than for any other pair-wise comparison. In the genome of a bacterium, the leading strand often has an excess G content compared with C, whereas the lagging strand has excess C compared with G [30, 31]. From the circular graph of GcM5-1A (**Fig 1**), the GC skew index (GC Skew = (G—C)/(G + C)) clearly demonstrated a G bias, with most of the GC skew indexes above 0 in the leading strand. C bias was observed in the lagging strand, which suggests that the established synteny was reliable and properly reflected the relationship between these *P*. *fluorescens* isolates.

### Candidate virulence factors

A previous study of the *P*. *fluorescens* GcM5-1A indicated that the isolate secreted *fliC* flagellin, which is deleterious to black pine seedlings [10]. When GcM5-1A and PWN were cultured together, the fecundity, egg-hatch rate and the development of PWN were significantly improved [32]. Xu *et al*. reported that treating the black pine cell suspension with the flagellin of GcM5-1A results in an oxidative burst of the pine cells and subsequent cell death, this indicating the host’s response to the attack of the pathogen [12]. However, the cell death of pine callus induced by flagellin promoted the proliferation of PWN and the associated GcM5-1A, which was feeding on the callus cells [11]. Li *et al*. further revealed that the flagellin of GcM5-1A would attach to the pine cell membrane and lead to shrinkage of the cell membrane, a concentration of the cytoplasm, formation of micronuclei, degradation of cytoplasmic RNA, and breakage of genomic DNA. However, DNA ladder formation was not observed, which indicated that an unusual form of apoptosis occurred in the flagellin-treated pine cells [33]. The above evidence suggested that GcM5-1A potentially plays a role in the pathogenesis of pine wilt disease.

Alignment of the N-terminal sequences of the *fliC* genes demonstrated that this gene shared high similarity between GcM5-1A and Pf-5 [12]. The expression the *fliC* genes of these two isolates in *Escherichia coli* produces recombinant flagellin that exhibit similar toxicity to pine suspension cells [10]. In a previous study, researchers observed that pine seedlings inoculated with the axenic PWN and transformed *E*. *coli* that produced secretive flagellin of Pf-5 resulted in symptoms of wilt. In contrast, pine seedlings inoculated with axenic PWN and untransformed *E*. *coli* did not cause symptoms of wilt [34]. Thus, the *fliC* gene is a crucial pathogenic factor in the genome of GcM5-1A and is located on contig57 in the genome assembly. Based on sequence similarity, we constructed a gene tree for the *fliC* genes in the five sequenced isolates of *P*. *fluorescens* (**Fig 4B**). The tree demonstrated that the *fliC* genes from Pf0-1 and Pf-5 were closely related, as were those of SBW25 and WH6. Whereas the *fliC* gene in GcM5-1A was more phylogenetically diverged from others, but it was more close to Pf0-1 and Pf-5 than SBW25 and WH6.

In addition to the *fliC* gene, we searched for additional virulence factors that might relate to pathogenesis in the genome of GcM5-1A. For this purpose, all of the predicted CDSs were submitted to BLAST against the VFDB, which was built for bacterial pathogens [35]. This analysis identified 79 CDSs as candidate virulence factors (**S1 Table**). Among these factors, the *Alg* genes encoding the polysaccharide alginate participate in the formation of biofilms, which play an important role in the plant-pathogen interaction [36]. The *Fl* genes encode the bacterial flagella that affect the bacterial motility. These genes are also essential for the formation of biofilms in *P*. *syringae* [37], whereas the *Waa* genes are involved in the synthesis and transformation of lipopolysaccharides in Gram-negative bacteria. Lipopolysaccharides play direct roles in the plant-pathogen interaction [38, 39]. The *Rhl* genes encode rhamno lipids that are crucial for *P*. *syringae* swarming, which is an important factor in pathogen infection [40]. All of the candidate virulence factors that were identified in the genome of GcM5-1A suggested that this microbe plays a role in the pathogenesis of pine wilt disease.

### The flagellum secretion system T3SS

**Fig 6:**
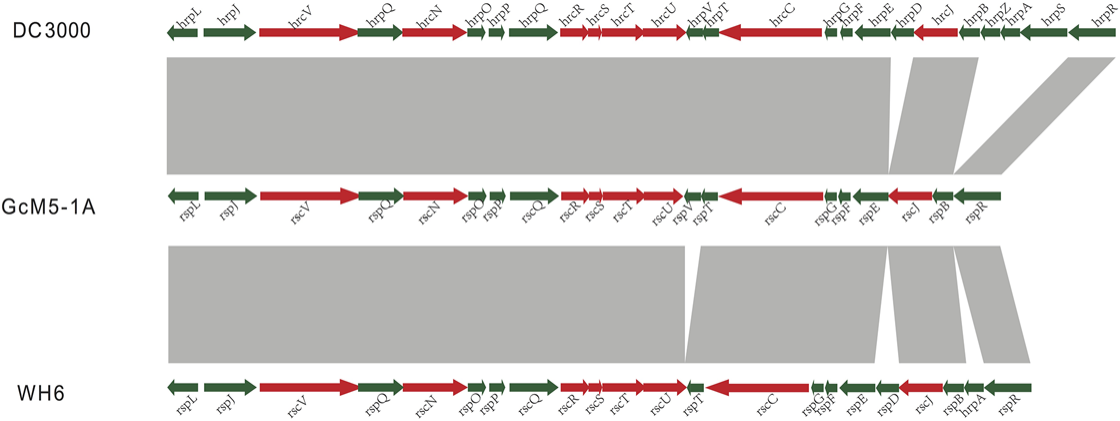
Comparisons of T3SS in GcM5-1A with that in *P*. *syringae* DC3000 and WH6.

In *Pseudomonas spp*., the complete T3SS was first identified in *P*. *syringae* pv. *tomato* DC3000. This microbe is the model system used to study T3SS, and it is pathogenic to tomato and *Arabidopsis* [41]. In pathogenic bacteria, T3SS uses its needle-like structure, known as the needle complex [42], to detect the presence of eukaryotic organisms. When eukaryotic organisms are detected, the T3SS injects bacterial proteins into the host cell and helps the bacteria colonize and multiply in the host cells [43, 44]. In the previously sequenced isolates of *P*. *fluorescens*, the complete T3SS was only identified in WH6. Using the T3SS genes from DC3000 and WH6 as queries, the complete T3SS was identified in the genome of GcM5-1A; thus, GcM5-1A appears to be the second *P*. *fluorescens* isolate that possesses the complete T3SS. In the GcM5-1A genome, CDS00691~CDS00712 were identified as T3SS genes (**Fig 6**). Similarity analysis of the T3SS candidates of GcM5-1A and DC3000 was performed by using BLASTP with 30% sequence identity and 80% sequence overlap. GcM5-1A was found to possess all the T3SS genes except for the D, Z, A, and S genes when compared with DC3000. GcM5-1A lacked D and A genes compared with WH6. In T3SS, the A and F genes were homologous to each other [45, 46]. The S and R genes shared high similarity and were functionally redundant [47]. Deletion of the Z gene did not affect T3SS function [48–50]. The function of the D gene remains unclear. Although upon consideration of the standard T3SS in DC3000, the genes that are lacking in GcM5-1A should have little effect on T3SS function. Based on the above analyses, we propose that the GcM5-1A genome possesses a fully functional T3SS.

## Conclusions

Pine wilt disease is a disastrous for pine forests, and the pathogenesis of this disease remains debatable. Some pathologists have proposed that the surface coat of PWN shelters microbes that might play a role in the onset of this disease. To provide insight into the pathogenicity of such microbes, we sequenced a Gram-negative bacterial isolate of *P*. *fluorescens*, GcM5-1A, which inhabits the surface coat of PWN. We obtained 1.5 million high-quality reads, which were assembled into sequence contigs 6.01 Mb in length. Sequence annotation predicated 5,413 CDSs. Among the five sequenced *P*. *fluorescens* isolates, GcM5-1A was more closely related to isolates SBW25 and WH6 than to Pf0-1 and Pf-5. We detected 79 virulence factors in the genome of GcM5-1A, including gene coding the major pathogenic protein *fliC*. GcM5-1A also possessed a fully functional T3SS. The detected candidate virulence factors provide informative clues to be addressed on in elucidating the role of GcM5-1A in the pathogenesis of pine wilt disease at molecular level in future studies. The availability of this genome sequence alongside that of PWN provides essential platforms for further study of their interaction and their roles in the pathogenesis of pine wilt disease.

## Materials and Methods

### Bacterial culture and genomic DNA extraction

*P*. *fluorescens* GcM5-1A (CCTCC No: M 204065) was isolated from PWN obtained from wilted *Pinus thunbergii* in Nanjing Forestry University, China. The isolation of GcM5-1A was performed as described by Zhao *et al*. [51]. Field studies were conduct under the permission of Nanjing Forestry University China. This isolate was shake-cultured in Luria-Bertani (LB) medium at 28°C for 24 h, and bacterial cells were harvested by centrifugation at 5,000×g for 10 min at 4°C. Genomic DNA of *P*. *fluorescens* GcM5-1A was isolated from the bacterial pellet according to the method described by Chen *et al*. [52]. DNA purity was examined by agarose gel electrophoresis, and the concentration was measured using a Nanodrop ND2000 (Thermo Scientific, Waltham, MA, USA).

### Sequencing and assembly

The GcM5-1A genome was sequenced on a 454 GS-FLX sequencer (Roche, Inc, Basel, Switzerland.) using the FLX Titanium Sequencing Kit (Roche, Inc, Basel, Switzerland.). Sequence assembly was conducted with a Newbler *de novo* Assembler (version 2.5.3) with default parameters. In the final assembly, contigs less than 100 bp in length were discarded. Sequence data from this study were deposited at DDBJ/EMBL/GenBank under accession number **JJOE00000000**.

### Gene annotation

The coding DNA sequences (CDSs) of GcM5-1A were predicted by Glimmer3 [53], which uses interpolated Markov models (IMMs) to identify the coding regions and distinguish them from noncoding DNA. Glimmer3 used the long-CDS program to find the long and non-overlapping CDSs, and then set the long-CDS training system for the subsequent predictions. The predicted genes were annotated against the nr database and the COG database, with an e-value cut-off of 1e-5.

### Non-coding RNA prediction

Transfer RNAs (tRNAs) were predicted using tRNAscan-SE [54]. This method first pre-filters the input sequence to identify candidate tRNAs; then, a highly selective tRNA “covariance model” is implemented for tRNA predictions and allows for the identification of 99 to 100% of tRNA genes in the DNA sequence with less than one false positive.

Ribosomal RNA (rRNA) was predicted using RNAmmer [55], which utilized two levels of hidden Markov models. An initial spotter mode searched both strands for detecting the approximate position of an rRNA gene. Flanking regions were then extracted and parsed to the full model, which matched the entire rRNA gene. By enabling this two-level approach, the program avoided running the entire genome sequence through the model and allowed faster predictions.

### Phylogenetic tree construction

Orthologous groups among the 5 isolates of *P*. *fluorescens* were distinguished using OrthoMCL, which utilized a Markov clustering algorithm to cluster the highly homologous genes from different isolates. By selecting the single-copy orthologous group, we first performed multiple alignment of each orthologous group on an amino acid level using MUSCLE (MUltiple Sequence Comparison by Log-Expectation). All multiple alignments were then combined into a multiple alignment or a so-called super-alignment file. By using the super-alignment file, the phylogenetic tree was constructed and visualized by MEGA5 using the neighbor-joining algorithm with a bootstrap support node set as r = 500. The construction of the phylogenetic tree of *fliC* genes was performed following the same method described above.

### Synteny and the circular graph

Synteny between GcM5-1A and the *P*. *fluorescens* isolates was performed by MUMmer [56] and visualized by *ad hoc* Perl scripts. The circular graph of GcM5-1A, which was consistent with the other four isolates of *P*. *fluorescens*, was constructed using DNAplotter [57].

### Identifying the T3SS and candidate virulence factors

To identify the T3SS in GcM5-1A, we submitted the T3SS genes of *P*. *syringae* pv. tomato DC3000 to BLAST against the entire gene set of GcM5-1A using BLASTP with parameters as follows: an e-value of 1e-5, 30% sequence similarity, 80% sequence length overlap and max_target_seqs of 1.

All of the predicted CDSs of GcM5-1A were submitted to BLAST against the Virulence Factor Database (VFDB) using BLASTP with an e-value of 1e-5 and max_target_seqs of 1. The BLAST results were then filtered using the criteria of sequence identity greater than 60% and sequence overlap greater than 90% between the query and the template.

## Supporting Information

S1 Table Candidate virulence factors identified in the genome of GcM5-1A. (XLS)

## References

[1] Haas D, Défago G. Biological control of soil-borne pathogens by fluorescent pseudomonads. Nat Rev Microbiol. 2005; 3: 307–319. 15759041 10.1038/nrmicro1129

[2] Li Y, Sun Z, Zhuang X, Xu L, Chen S, Li M. Research progress on microbial herbicides. Crop Prot. 2003; 22: 247–252.

[3] Paulsen IT, Press CM, Ravel J, Kobayashi DY, Myers GSA, Mavrodi DV, et al Complete genome sequence of the plant commensal *Pseudomonas fluorescens* Pf-5. Nat Biotechnol. 2005; 23: 873–878. 15980861 10.1038/nbt1110 PMC7416659

[4] Kimbrel JA, Givan SA, Halgren AB, Creason AL, Mills DI, Banowetz GM, et al An improved, high-quality draft genome sequence of the Germination-Arrest Factor-producing *Pseudomonas fluorescens* WH6. BMC Genomics. 2010; 11: 522 10.1186/1471-2164-11-522 20920191 PMC2997014

[5] Silby MW, Cerdeno-Tarraga AM, Vernikos GS, Giddens SR, Jackson RW, Preston GM, et al Genomic and genetic analyses of diversity and plant interactions of *Pseudomonas fluorescens* . Genome Biol. 2009; 10: R51 10.1186/gb-2009-10-5-r51 19432983 PMC2718517

[6] Trippe K, McPhail K, Armstrong D, Azevedo M, Banowetz G. *Pseudomonas fluorescens* SBW25 produces furanomycin, a non-proteinogenic amino acid with selective antimicrobial properties. BMC microbiol. 2013; 13: 111 10.1186/1471-2180-13-111 23688329 PMC3662646

[7] Halgren A, Maselko M, Azevedo M, Mills D, Armstrong D, Banowetz D. Genetics of germination-arrest factor (GAF) production by *Pseudomonas fluorescens* WH6: identification of a gene cluster essential for GAF biosynthesis. Microbiology. 2013; 159: 36–45. 10.1099/mic.0.062166-0 23125119

[8] Zhao BG, Liu YF. Effects of Bacteria Associated with Pine Wood Nematode (*Bursaphelenchus xylophilus*) on Development and Egg Production of the Nematode. J Phytopathol. 2007; 155:26–30.

[9] Shinya R, Morisaka H, Takeuchi Y, Ueda M, Futai K. Comparison of the surface coat proteins of the pine wood nematode appeared during host pine infection and in vitro culture by a proteomic approach. Phytopathol. 2010; 100: 1289–1297. 10.1094/PHYTO-04-10-0109 21062170

[10] Guo D, Zhao B, Li R, Kulinch Q, Ryss A. Purification of flagellin of *Pseudomonas fluorescens* GcM5-1A carried by the pine wood nematote, *Bursaphelenchus xylophilus*, and its in vitro toxicity to a suspension of cells of *Pinus thunbergii* . Russ J Nematol. 2008; 16:151–157.

[11] Zhang L, Yue T, Zhao B, Guo D, Wu B, Wang T, et al Flagellin promotes propagation of pine wood nematode and its carrying Pseudomonas fluorescens GcM5-1A in callus of *Pinus thunbergii* through inducing cell death. Afr J Microbiol Res. 2012; 6: 1322–1328.

[12] Xu Z, Yu J, Cui L, Li M, Li R, Guo D. Effects of *Pseudomonas fluorescens* flagellin on physiological and biochemical characteristics in the suspension cells of *Pinus thunbergii* . Eur J Plant Pathol. 2013; 136: 729–736.

[13] Taisei K, Cotton JA, Dalzell JJ, Hasegawa K, Kanzaki N, McVeigh P, et al Genomic insights into the origin of parasitism in the emerging plant pathogen Bursaphelenchus xylophilus. Plos Pathogens. 2011; 7:e1002219–e1002219. 10.1371/journal.ppat.1002219 21909270 PMC3164644

[14] Filipiak A. The pine wilt disease. Sylwan 2008; 152: 9–19.

[15] Suzuki K. Pine wilt disease–a threat to pine forest in Europe. Dendrobiology. 2002; 48:71–74.

[16] Mamiya Y. Pathology of the pine wilt disease caused by *Bursaphelenchus xylophilus* . Annu Rev Phytopathol. 1983; 21: 201–220. 10.1146/annurev.py.21.090183.001221 25946434

[17] Ryss AY, Kulinich OA, Sutherland JR. Pine wilt disease. a short review of worldwide research. Forestry Studies in China. 2011; 13: 132–138.

[18] Nascimento FX, Hasegawa K, Mota M, Vicente CS. Bacterial role in pine wilt disease development–review and future perspectives. Env Microbiol Rep. 2015; 7:51–63. 25139220 10.1111/1758-2229.12202

[19] Daniela B, Sheng YH. Type III Protein Secretion in Plant Pathogenic Bacteria. Plant Physiol. 2009; 150:1656–1664. 10.1104/pp.109.139089 19458111 PMC2719110

[20] Coburn B, Sekirov I, Finlay BB. Type III secretion systems and disease. Clin Microbiol Rev. 2007; 20: 535–549. 17934073 10.1128/CMR.00013-07 PMC2176049

[21] Banowetz GM, Azevedo MD, Armstrong DJ, Halgren AB, Mills DI. Germination-Arrest Factor (GAF): Biological properties of a novel, naturally-occurring herbicide produced by selected isolates of rhizosphere bacteria. Biol Control. 2008; 46: 380–390.

[22] Altschul SF, Madden TL, Schäffer AA, Zhang J, Zhang Z, Miller W, et al Gapped BLAST and PSI-BLAST: a new generation of protein database search programs. Nucleic Acids Res. 1997; 25: 3389–3402. 9254694 10.1093/nar/25.17.3389 PMC146917

[23] Rissman AI, Mau B, Biehl BS, Darling AE, Glasner JD, Perna NT. Reordering contigs of draft genomes using the Mauve aligner. Bioinformatics. 2009; 25: 2071–2073. 10.1093/bioinformatics/btp356 19515959 PMC2723005

[24] Chen F, Mackey AJ, Stoeckert CJ Jr, Roos DS. OrthoMCL-DB: querying a comprehensive multi-species collection of ortholog groups. Nucleic Acids Res. 2006; 34: D363–368. 16381887 10.1093/nar/gkj123 PMC1347485

[25] Tatusov RL, Galperin MY, Natale DA, Koonin EV. The COG database: a tool for genome-scale analysis of protein functions and evolution. Nucleic Acids Res. 2010; 28: 33–36. 10.1093/nar/28.1.33 PMC102395 10592175

[26] Feil H, Feil WS, Chain P, Larimer F, DiBartolo G, Copeland A, et al Comparison of the complete genome sequences of *Pseudomonas syringae* pv. syringae B728a and pv. tomato DC3000. Proc Natl Acad Sci. 2005; 102: 11064–11069. 16043691 10.1073/pnas.0504930102 PMC1182459

[27] Mathee K, Narasimhan G, Valdes C, Qiu X, Matewish JM, Koehrsen M, et al Dynamics of *Pseudomonas aeruginosa* genome evolution. Proc Natl Acad Sci. 2008; 105: 3100–3105. 10.1073/pnas.0711982105 18287045 PMC2268591

[28] Edgar RC. MUSCLE: multiple sequence alignment with high accuracy and high throughput. Nucleic Acids Res. 2004; 32: 1792–1797. 15034147 10.1093/nar/gkh340 PMC390337

[29] Tamura K, Peterson D, Peterson N, Stecher G, Nei M, Kumar S. MEGA5: molecular evolutionary genetics analysis using maximum likelihood, evolutionary distance, and maximum parsimony methods. Mol Biol Evo. 2011; 28: 2731–2739. 10.1093/molbev/msr121 PMC3203626 21546353

[30] Arakawa K, Tomita M. The GC Skew Index: A Measure of Genomic Compositional Asymmetry and the Degree of Replicational Selection. Evol Bioinform. 2007; 3: 159–168. PMC2684130 19461976

[31] Marin A, Xia XH. GC skew in protein-coding genes between the leading and lagging strands in bacterial genomes: New substitution models incorporating strand bias. J Theor Biol. 2008; 253: 508–513. 10.1016/j.jtbi.2008.04.004 18486155

[32] Zhao B, Liu Y, Lin F. Mutual influences between *Bursaphelenchus xylophilus* and bacteria carries. J Nanjing Forestry Univ (Nat Sci Ed). 2005; 29:1–4.

[33] Li S, Guo D, Zhao B, LI R. Lethal effect of flagellin secreted by *Pseudomonas fluorescens* on cells of *Pinus thunbergii* . Acta Bot Boreali-Occidential Sinica. 2008; 28: 2154–2158.

[34] Li S, Zhao B, Li R, Guo D. Construction of engineering bacterium expressing flagellin of *Pseudomonas fluorescens* and its toxicity to *Pinus thunbergii in vivo* . J Qingdao Univ (Nat Sci Ed). 2010; 25: 35–40.

[35] Yang J, Chen L, Sun L, Yu J, Jin Q. VFDB 2008 release: an enhanced web-based resource for comparative pathogenomics. Nucleic Acids Res. 2008; 36: D539–542. 17984080 10.1093/nar/gkm951 PMC2238871

[36] Gacesa P. Bacterial alginate biosynthesis-recent progress and future prospects. Microbiology. 1998; 144: 1133–1143. 9611788 10.1099/00221287-144-5-1133

[37] O'Toole GA, Kolter R. Flagellar and twitching motility are necessary for Pseudomonas aeruginosa biofilm development. Mol Microbiol. 2002: 30: 295–304. 10.1046/j.1365-2958.1998.01062.x 9791175

[38] Newman MA, Dow J, Daniels M. Bacterial lipopolysaccharides and plant-pathogen interactions. Eur J Plant Pathol. 2001; 107: 95–102.

[39] Conrath U, Pieterse CMJ, Mauch-Mani B. Priming in plant-pathogen interactions. Trends Plant Sci. 2002; 7: 210–216. 11992826 10.1016/s1360-1385(02)02244-6

[40] Xu J, Platt TG, Fuqua C. Regulatory linkages between flagella and surfactant during swarming behavior: lubricating the flagellar propeller? J Bacteriol. 2012; 194: 1283–1286. 10.1128/JB.00019-12 22267512 PMC3294858

[41] Blocker A, Jouihri N, Larquet E, Gounon P, Ebe F, Parsot C, et al Structure and composition of the Shigella flexneri ‘needle complex’, a part of its type III secreton. Mol Microbiol. 2001; 39: 652–663. 11169106 10.1046/j.1365-2958.2001.02200.x

[42] Ghosh P. Process of protein transport by the type III secretion system. Mol Biol Rev. 2004; 68: 771–795. 10.1128/MMBR.68.4.771-795.2004 PMC539011 15590783

[43] Gophna U, Ron EZ, Graur D. Bacterial type III secretion systems are ancient and evolved by multiple horizontal-transfer events. Gene. 2003; 312: 151–163. 12909351 10.1016/s0378-1119(03)00612-7

[44] Buell CR, Joardar V, Lindeberg M, Selengut J, Paulsen IT, Gwinn ML, et al The complete genome sequence of the *Arabidopsis* and tomato pathogen *Pseudomonas syringae* pv. *tomato* DC3000. Proc Natl Acad Sci. 2003; 100: 10181 12928499 10.1073/pnas.1731982100 PMC193536

[45] Deng WL, Preston G, Collmer A, Chang CJ, Huang HC. Characterization of the hrpC and hrpRS operons of *Pseudomonas syringae* pathovars syringae, tomato, and glycinea and analysis of the ability of hrpF, hrpG, hrcC, hrpT, and hrpV mutants to elicit the hypersensitive response and disease in plants. J Bacteriol. 1998; 180: 4523–4531. 9721291 10.1128/jb.180.17.4523-4531.1998 PMC107463

[46] Lee YH, Kolade OO, Arvidson DN, He SY. Identification of HrpA mutants that block type III secretion in *Pseudomonas syringae* pv. *tomato* DC3000. Phytopathology. 2004; 94: S59–S59.

[47] Wei Z, Kim JF, Beer SV. Regulation of hrp genes and type III protein secretion in *Erwinia amylovora* by HrpX/HrpY, a novel two-component system, and HrpS. Mol Plant Microbe In. 2000; 13: 1251–1262. 10.1094/MPMI.2000.13.11.1251 11059492

[48] Alfano JR, Bauer DW, Milos TM, Collmer A. Analysis of the role of the *Pseudomonas syringae* pv. *syringae* HrpZ harpin in elicitation of the hypersensitive response in tobacco using functionally non-polar hrpZ deletion mutations, truncated HrpZ fragments, and hrmA mutations. Mol Microbiol. 1996; 19: 715–728. 8820642 10.1046/j.1365-2958.1996.415946.x

[49] Ortiz-Martin I, Thwaites R, Mansfield JW, Beuzon CR. Negative regulation of the Hrp type III secretion system in *Pseudomonas syringae* pv. *phaseolicola* . Mol Plant Microbe In. 2010; 23: 682–701. 10.1094/MPMI-23-5-0682 20367475

[50] Preston G, Deng WL, Huang HC, Collmer A. Negative regulation of hrp genes in *Pseudomonas syringae* by HrpV. J Bacteriol. 1998; 180: 4532–4537. 9721292 10.1128/jb.180.17.4532-4537.1998 PMC107464

[51] Zhao BG, Wang HL, Han SF, Han ZM. Distribution and pathogenicity of bacteria species carried by Bursaphelenchus xylophilus in China. Nematology. 2003; 5:899–906.

[52] Chen WP, Kuo TT. A simple and rapid method for the preparation of gramnegative bacterial genomic DNA. Nucleic Acids Res. 1993; 21: 2260 8502576 10.1093/nar/21.9.2260 PMC309503

[53] Delcher AL, Bratke KA, Powers EC, Salzberg SL. Identifying bacterial genes and endosymbiont DNA with Glimmer. Bioinformatics. 2007; 23: 673–679. 17237039 10.1093/bioinformatics/btm009 PMC2387122

[54] Schattner P, Brooks AN, Lowe TM. The tRNAscan-SE, snoscan and snoGPS web servers for the detection of tRNAs and snoRNAs. Nucleic Acids Res. 2005; 33: W686–W689. 15980563 10.1093/nar/gki366 PMC1160127

[55] Lagesen K, Hallin P, Rodland EA, Staerfeldt HH, Rognes T, Ussery DW. RNAmmer: consistent and rapid annotation of ribosomal RNA genes. Nucleic Acids Res. 2007; 35: 3100–3108. 17452365 10.1093/nar/gkm160 PMC1888812

[56] Kurtz S, Phillippy A, Delcher AL, Smoot M, Shumway M, Antonescu C, et al Versatile and open software for comparing large genomes. Genome Biol. 2004; 5: R12 14759262 10.1186/gb-2004-5-2-r12 PMC395750

[57] Carver T, Thomson N, Bleasby A, Berriman M, Parkhill J. DNAPlotter: circular and linear interactive genome visualization. Bioinformatics. 2009; 25: 119–120. 10.1093/bioinformatics/btn578 18990721 PMC2612626

